# Effects of Copper and/or Cholesterol Overload on Mitochondrial Function in a Rat Model of Incipient Neurodegeneration

**DOI:** 10.1155/2013/645379

**Published:** 2013-11-06

**Authors:** Nathalie Arnal, Omar Castillo, María J. T. de Alaniz, Carlos A. Marra

**Affiliations:** ^1^Instituto de Investigaciones Bioquímicas de La Plata (INIBIOLP), CCT La Plata, CONICET-UNLP, Cátedra de Bioquímica y Biología Molecular, Facultad de Ciencias Médicas, Universidad Nacional de La Plata, 60 y 120, 1900 La Plata, Argentina; ^2^Centro de Investigaciones Cardiovasculares (CIC), CCT-CONICET, 1900 La Plata, Argentina; ^3^INIBIOLP, Cátedra de Bioquímica, Facultad de Ciencias Médicas, Universidad Nacional de La Plata, Calles 60 y 120, 1900 La Plata, Argentina

## Abstract

Copper (Cu) and cholesterol (Cho) are both associated with neurodegenerative illnesses in humans and animals models. We studied the effect in Wistar rats of oral supplementation with trace amounts of Cu (3 ppm) and/or Cho (2%) in drinking water for 2 months. Increased amounts of nonceruloplasmin-bound Cu were observed in plasma and brain hippocampus together with a higher concentration of ceruloplasmin in plasma, cortex, and hippocampus. Cu, Cho, and the combined treatment Cu + Cho were able to induce a higher Cho/phospholipid ratio in mitochondrial membranes with a simultaneous decrease in glutathione content. The concentration of cardiolipin decreased and that of peroxidation products, conjugated dienes and lipoperoxides, increased. Treatments including Cho produced rigidization in both the outer and inner mitochondrial membranes with a simultaneous increase in permeability. No significant increase in Cyt C leakage to the cytosol was observed except in the case of cortex from rats treated with Cu and Cho nor were there any significant changes in caspase-3 activity and the Bax/Bcl2 ratio. However, the A**β**(1–42)/(1–40) ratio was higher in cortex and hippocampus. These findings suggest an incipient neurodegenerative process induced by Cu or Cho that might be potentiated by the association of the two supplements.

## 1. Introduction

It is well known that copper (Cu) is an essential transition metal for all living organisms, functioning as cofactor for many enzymes [1–3]. However, we and other laboratories have demonstrated *in vivo *and *in vitro* that excess inorganic Cu produces increased levels of reactive oxygen species (ROS) and damage to biomolecules, ultimately promoting cell death [4–7]. Humans are continuously at risk from excess Cu due to involuntary exposure to pollution (contaminated water, food), professional activities [8–10], ingestion of dietary supplements [10–12], and prolonged use of intrauterine devices [13, 14]. Elevated Cu plasma levels, especially of free-Cu or the so-called nonceruloplasmin-bound Cu (NCBC), have been associated with neurodegenerative damage [10, 15, 16]. In recent years, there has been a considerable increase in the number of published papers relating Cu to the neurodegenerative process [15–18]. In line with this, Brewer [10] hypothesized that ingestion of inorganic Cu from different sources is at least a partial cause of Alzheimer disease (AD) in developed countries. Squitti et al. [17–19] reported that NCBC, which is loosely bound to molecules such as serum albumin and other proteins, is one of the main risk factors involved in AD development. They suggest that it is the ratio of CP to Cu that is the crucial biomarker for interpreting Cu-associated features in live AD patients.

AD has multiple conditioning and causative factors. However, it was reported that inorganic Cu in conjunction with a high fat diet sets the stage for the development of AD, particularly if other risk factors are present [19]. Pappolla et al. [20] proposed that hypercholesterolemia accelerates the biochemical neuropathologic damage observed in transgenic mice. In addition, Notkola et al. [21] found that plasma cholesterol (Cho) levels were predictors of AD prevalence in a population-based sample of 444 men (aged 70–89 years). More recently, *in vivo* models of AD-like neurodegenerative diseases demonstrated that the association of Cu and Cho increases the risk of the development of neurodegenerative damage [22–24]. 

Cu overload has been extensively associated with ROS overproduction *in vitro* and *in vivo* [6, 7, 25]. Oxidative stress is considered a primary event in the development of AD [26]. Pope et al. [27] reported that ROS overproduction due to the dysfunction of the electron transport chain is causative of neurodegeneration associated with AD and Parkinson's disease. Moreover, some experimental evidence indicates that alterations in mitochondrial energetics occur along with the accumulation of oxidative damage before the cardinal signs of AD pathogenesis in the brains of transgenic animal models and human patients [28, 29].

As reviewed by Paradies et al. [30], the mitochondrion is considered the most important cellular organelle contributing to the neurodegenerative process, mainly through respiratory chain dysfunction and the formation of reactive oxygen species. Factors that increase the rate of mitochondrial ROS production lead to mitochondrial dysfunction because of the oxidative-induced damage caused to various crucial biomolecules such as mtDNA, lipids, and proteins, thus increasing mitochondrial deterioration in a self-perpetuating process [31]. Together with ROS accumulation, mitochondrial dysfunction is one of the earliest and most prominent features of AD [27, 28] and this condition is strongly associated with multiple negative consequences. Dysfunctional mitochondria generate high levels of ROS that are ultimately toxic to cells, particularly those with a long lifespan and low antioxidant defense system such as neurons [28, 29]. At the same time, mitochondria are also targets of the ROS produced by pro-oxidative factors such as Cu overload or increased levels of NCBC.

Among the mitochondrial lipids directly affected by ROS overproduction, cardiolipin (CL) is one of the most deeply involved in both synapses and neuronal loss [31]. CL is linked to the endogenous proapoptotic cascade that eventually leads to the integration of the apoptosome and the subsequent activation of the effector caspase system [28–31]. Moreover, CL is involved in the regulation of the bioenergetic process and in the integrity of mitochondrial membranes. Peroxidation of CL directly affects the biochemical functions that depend on the inner and outer mitochondrial membrane composition and structure. Experimental evidence clearly indicates that this lipid represents the most important target of ROS attack [30].

In summary, there is conclusive evidence linking ROS hyperproduction with mitochondrial dysfunction. Oxidative stress is also directly involved in neurodegeneration and Cu overload in association with cholesterol apparently acts as a synergistic risk factor in the etiology of neurological disorders. All the foregoing evidence suggests the need for more in-depth study of this phenomenon. In view of the absence of conclusive studies on the biochemical effects of the Cu + Cho (CuCho) association on mitochondrial-associated dysfunction, especially in relation to those aspects involved in programmed cell death, we aimed to investigate the effects of CuCho on mitochondria isolated from cortex and hippocampus of Wistar rats and explore (i) the Cho/Pi ratio and steady-state fluorescence anisotropy of the outer and inner mitochondrial membranes in order to assess their fluidities; (ii) mitochondrial membrane integrity by means of a probe that measures trans-membrane potential; (iii) the concentration of the main mitochondrial antioxidant glutathione; (iv) the concentration of the key mitochondrial phospholipid, cardiolipin, and the possible pro-oxidative damage it causes; (v) the activity of the apoptotic pathway depending on mitochondrial membrane integrity (caspase-3 and the Bax/Bcl-2 ratio); and (vi) the concentration of *β*-amyloid (A*β*) in the two brain zones and in peripheral plasma as a biomarker of a proneurodegenerative effect.

## 2. Materials and Methods

### 2.1. Chemicals

All chemicals used were of analytical grade and obtained from Sigma Chem. Co. (Buenos Aires, Argentina or USA), Merck (Darmstadt, Germany), and Carlo Erba (Milan, Italy).

### 2.2. Animals and Treatments

Certified pathogen-free male Wistar rats were used. The rats were maintained at a controlled temperature (25°C) and relative humidity of 60% with forced ventilation, under a normal photoperiod of 12 h darkness and 12 h light. The health of the animals was monitored in accordance with the internationally recommended practices of the *Institute of Laboratory Animal Resources, Commission of Life Sciences, National Research Council* (ILAR). Solid food and drinking water were provided *ad libitum*. The diets for the experiments were prepared in our laboratory according to the recommendations for Wistar rats [32]. All procedures for handling the animals followed the NIH regulations [33]. The experimental protocol was reviewed and approved by the Bioethics Committee of the Faculty of Medical Sciences, UNLP (number 00382/11).

### 2.3. Experimental Protocols

Rats (21 days old) were randomly assigned (ten animals per group) to the protocols detailed as follows and treated for eight weeks (2 months). Selecting very young rats lets us discard any neurodegenerative (unknown) event(s) associated to the age of the animal. By the way, this approach may be extrapolated to humans that are exposed to copper from the very beginning of their life. In addition, in previous experiments (not shown), we noted that—at lest during the first year of life—that we have no differences in the response of the model as a function of the starting age of treatment. The control group (C) was maintained on lab-prepared pellets as recommended for normal growth containing 7 ppm of Cu [34, 35]. The Cu-supplemented experimental group (Cu) was fed on control pellets and tap water supplemented with 3 mg/L (or ppm) of Cu in the form of ultrapure CuSO_4_ (Merck, Darmstadt, Germany). The Cho-supplemented group (Cho) was fed on pellets containing 2% (W/W) of Cho (87% pure) (obtained from Saporiti SRL, Buenos Aires, AR), and the Cu + Cho-supplemented group (CuCho) was simultaneously treated with Cu in water + Cho in food. Rats were monitored during the experimental period to observe their behavior, quantify water and food consumption, and determine their body weight gain. Total Cu concentration in tap water supplemented with CuSO was determined by means of atomic absorption methodology and was 3.42 ± 0.21 ppm (means of all daily measurements taken along the experimental period). Considering that each animal imbibed between 4.9 ± 0.4 and 15.0 ± 1.1 mL water/day (at the beginning and the end of the protocol, resp.), a maximum of 0.01 to 0.05 mg Cu/day was acquired from water (a dose equivalent to 0.06 and 0.18 mg Cu/Kg live animal, resp.). Linear regression curves and ANOVA test for Cu content in food demonstrated that there were no significant variations between the 6 preparations used for the experiments (7.22 ± 0.31 ppm or mg Cu/Kg diet). Fe and Zn content (determined by atomic absorption spectrometry) was the same in all preparations (45.9 ± 0.8 and 66.6 ± 2.0 ppm, resp.). Ingestion of solid food along the experiments varied from 11.6 ± 0.8 to 29.7 ± 2.8 g/rat, implying that the oral ingestion of Cu was in the range from 0.08 to 0.21 mg Cu/day/rat (0.0032 to 0.0008 mg Cu/Kg live animal).

### 2.4. Sample Collection

At the end of the treatments, animals were deeply anesthetized with ketamine (70 mg/Kg) and xylazine (5 mg/Kg) applied intramuscularly and then sacrificed by decapitation. Brains were removed and dissected into two zones: cortex and hippocampus, using the atlas of Paxinos and Watson [36] as a guide for tissue dissection. Both brain regions were washed, weighed, and homogenized using a Tris/HCl buffer (10 mM pH 7.4) with sucrose (70 mM), mannitol (230 mM), ethylenediaminetetraacetic acid (EDTA) (1 Mm), and dithiothreitol (DTT) (1 mM) (Buffer I). Homogenates were centrifuged at 2°C 700 ×g for 10 min and the supernatants recentrifuged at 8,000 ×g (10 min/2°C) to obtain the cytoplasm fraction. Pure mitochondrial fractions were isolated from the pellets of the previous centrifugation by resuspending them in 3 mL of buffer I and centrifuging during 5 min at 1,000 ×g (2°C). The pellets were discarded and the supernatants re-centrifuged for 10 min at 11,000 ×g (2°C). Finally, the final pellet (mitochondrial fraction) was resuspended in buffer II (buffer I with 0.015% mg pure albumin (Sigma Chem. Co.; Buenos Aires, AR)). Cytosol fractions were prepared by ultracentrifugation of the supernatants at 110,000 ×g for 1 h at 2°C. During the sacrifice of the animals, blood was also collected using heparin as anticoagulant (1 IU/5 mL) in ice-cold polypropylene tubes. The plasma samples were immediately prepared by centrifugation in the cold (4,000 ×g, 10 min) and stored at −70°C until analyzed.

### 2.5. Atomic Absorption Measurements

Aliquots of sample were digested with a mixture of 4 mL of HNO_3_ (c) and 1 mL HClO_4_ (Aldrich or Sigma Chem. Co., Buenos Aires, Argentina) by heating at 120°C for 60 min in a mineralization block [37]. The digests were cooled, diluted with ultrapure water (18 mΩ cm, Carlo Erba, Milan, Italy), and ultrafiltered with a 0.22 *μ*m Millipore membrane (Milli-Q Purification System, from Millipore, CA, USA). Ultrafiltered dissolutions were directly aspirated into the flame of a PerkinElmer 1100 B Spectrophotometer equipped with a Perkin-Elmer cathode lamp (Perkin-Elmer Corp., Norwalk, CT, USA) at a spectral width of 1 nm. Standard solutions of 100 ppm Zn and Fe from HCR Inc. (QuimiNet, Buenos Aires, AR) were used. Cu determinations were calibrated with a standard solution (200 ppm) of Cu(NO_3_)_2_ in HNO_3_ 0.5 N (Titrisol from Merck Co., Darmstadt, Germany). All measurements were carried out in peak height mode (324.7 nm line). The intra-[(SD/x) · 100] and inter-[(ΔSD/Δx) · 100] assay coefficients of variation were 15.5 and 6.0%, respectively. We routinely obtained a similar equation for the calibration curve (IR = 55 ·  10^−5^ + 0.048 [Cu, mg/L]), and the statistical analyses demonstrated a correlation coefficient always between 0.95 and 0.99. In addition, we explored the so-called matrix effects that might have modified the slopes of the standard regressions. In spiked samples, the obtained values varying from 48 to 60 · 10^−5^ were very similar to those of Cu standard solutions, indicating that the matrix effect was considered nonsignificant or was negligible. The mean for recovery and RSD for spiked samples was 99.7% and 3.3%, respectively, and the detection limit was 0.09 mg/L. In order to verify the accuracy of the method, we explored the influence of time after dilution, temperature of acid digestion, and concentration of HNO_3_/HClO_4_ following the suggestions of Terrés-Martos et al. [38]. We also checked our results with biological samples (plasma and homogenates) against a Seronorm Trace Elements Serum (from Sero Labs, Billingstad, Norway) and found no significant differences between the obtained and the declared (certified) concentrations.

### 2.6. Ceruloplasmin (CP) Levels and Nonceruloplasmin-Bound Copper (NCBC)

Samples were analyzed by the enzyme conversion of p-phenylenediamine into a blue-colored product [39] which was then measured at 550 nm. Reaction proceeded at 37°C in buffer glacial acetic/sodium acetate (50 mM, pH 5.5) directly into flat-bottomed plates, using a Microplate Reader SpectraMax M2/m2^e^ model from Molecular Devices Analytical Technologies (Sunnyvale, CA, USA) for 3 min. Intra- and interassay coefficients of variation were 8.3 and 4.4%, respectively. CP concentrations were calculated by comparison with the reaction rate of pure CP standard (Sigma Chem. Co., Buenos Aires, Argentina). Using the Cu and CP data we calculated the non-CP-bound Cu (NCBC, or so-called free Cu) as described by Brewer [19] by subtracting the amount of Cu bound to each mg of CP from data of total Cu. This parameter can be easily expressed in percentages using the formula (([Cu] − 47.2 × [CP]) × 100/[Cu]) where Cu is in *μ*mol/L and CP in g/L [40].

### 2.7. Lipid Analysis

Total lipids were extracted by the method of Folch et al. [41]. An aliquot of each Folch extract was evaporated and the residue dissolved in 50 mM sodium phosphate buffer (pH 7.4) containing digitonin 1%. Aliquots of this solution were taken to enzymatically measure cholesterol (Cho) and phospholipids (PL) using commercial kits from Wienner Lab (Rosario, Argentina). To determine the mitochondrial cardiolipin (CL) content, samples were separated by high-performance thin-layer chromatography (HPTLC) on precoated silica gel plates (10 × 20 cm) with concentration zone from Whatman Schleicher and Schuell (Maidstone, England). The mobile phase was chloroform : methanol : ammonium hydroxide (65 : 25 : 4; by volume). Spots were localized using iodine vapor on a lateral lane spotted with authentic standards of CL, mono- and dilyso-CL, and other reference phospholipids (Avanti Polar Lipids, Ontario, Canada). The rest of the plate was covered with a glass to avoid possible spontaneous oxidation. The CL and lyso-CL zones were scraped off the plates and eluted from the silica with “inverse” Folch extraction (chloroform : methanol; 1 : 2). After evaporation under nitrogen at room temperature, the selected zones were dissolved in 200 *μ*L of Folch solvent mixture. The total amount of CL and lyso-CL was quantified by means of phosphorous measurement using the method of Chen et al. [42]. 

### 2.8. Mitochondrial Membrane Integrity

The mitochondrial membrane potential (Δ*ψ*) was measured by estimating the integrity of the mitochondrial membrane using the isolated mitochondrial staining kit from Sigma-Aldrich, Inc. (Buenos Aires, AR). This kit is based on the uptake of the lipophilic cationic probe 5,5′,6,6′-tetrachloro-1,1′,3,3′-tetraethyl-benzimidazolcarbocyanine iodide dye (JC-1) into the mitochondrial matrix which directly depends on the Δ*ψ*. In healthy cells, the dye concentrates in the matrix, where it forms bright red fluorescent agglomerates. Uptake by the mitochondria of JC-1 can be utilized as an effective distinction between apoptotic and healthy cells. Any event that dissipates the Δ*ψ* prevents the accumulation of the JC-1 dye in the mitochondria, and the dye is thus dispersed in the cytoplasm, leading to a shift from red (JC-1 agglomerated) to green fluorescence (JC-1 monomers). To measure the samples, we used a PerkinElmer LS 55 Fluorescence Spectrometer, set at 525 nm excitation wavelength and 590 nm emission.

### 2.9. Steady-State Fluorescence Anisotropy of DPH and TMA-DPH

Submitochondrial fractions (inner and outer membranes) were obtained following the method described by Fraser and Zammit [43] with the modifications of Pellon-Maison et al. [44]. Prior to membrane fluidity assays, total protein concentration was determined in each suspension and adjusted to approximately 300 *μ*g protein/mL using 5 mM TRIS/HCL buffer pH 7.40. Membrane fluidity was determined by the fluorescence anisotropy (or polarization) technique using two fluorescent probes: 1,6-diphenyl-1,3,5-hexatriene (DPH; Sigma-Aldrich, St. Louis, MO) and 1-[4-(trimethylamino)phenyl]-6-phenyl-1,3,5-hexatriene (TMA-DPH; Molecular Probes, Eugene, OR). TMA-DPH was used to monitor fluidity near the surface of the cell membrane. The polar region of this probe is anchored at the lipid-water interface and the hydrocarbon moiety enters the lipid part of the membrane. The length of the hydrophobic part of the TMA-DPH molecule is approximately equivalent to that of a plasma membrane [45]. DPH is incorporated into the hydrophobic regions of the lipid bilayer [46]. DPH or TMA-DPH anisotropy is inversely correlated with membrane fluidity [47]. Anisotropy measurements were performed as previously described [48], with slight modifications. Briefly, membranes were vigorously homogenized using a vortex vibrator for 1 min and subjected to mild sonication in a Branson sonifier 450 (Branson Ultrasonic SA, CA, USA) (set at 40% output) following a 3 min incubation period on ice. The sonication cycle was controlled by the turbidity of the suspension as evaluated at 600 nm and was interrupted when an absorbance value of 0.2 or less was reached. This procedure does not interfere with the transition of lipid bilayers but disperses aggregates, facilitating fluorescence readings and decreasing light scattering [49]. The samples for the DPH and TMA-DPH assays (3 mL) contained 5 mM Tris–HCl buffer pH 7.4, 60 *μ*g of protein from diluted suspensions, and 50 *μ*M MDPH in tetrahydrofuran or 25 *μ*M TMA-DPH in dimethylformamide and were incubated for 45 min at 37°C. The steady-state anisotropy was measured in a He*λ*10S*β* spectrofluorophotometer (Thermoelectron Corp., Sydney, Australia) equipped with a thermostated cell holder. The sample's temperature was checked to an accuracy of ±0.1°C using a thermistor thermometer. Excitation and emission were set at 357/435 nm and 358/428 nm for DPH and TMA-DPH (resp.) using 5 nm excitation and emission slits. Samples were illuminated with linear (vertically, *v*, or horizontally, *h*) polarized monochromatic light and the fluorescence intensities emitted (*I*, in arbitrary units) parallel or perpendicular to the direction of the excitation beam were recorded. Blank samples without the addition of the probe(s) were also measured to estimate unspecific light that might reach the detector. The fluorescence anisotropy of the probes (*X*) was calculated as *r*(*X*) = [*Ivv* − *Ivh* × *G*] = [*Ivv* + 2*Ivh* × *G*], where *Ivv* and *Ivh* are the intensities of the fluorescence emitted, respectively, parallel and perpendicular to the direction of the vertically polarized excitation light; G is the correction factor (*G* = *Ihv*/*Ihh*) for the optical system given by the ratio of the vertically to the horizontally polarized emission components when the excitation light is polarized in the horizontal direction; and *X* represents TMA-DPH or DPH. According to Shinitzky and Barenholz [50], fluorescence anisotropy values are inversely proportional to cell membrane fluidity. Thus, a high degree of fluorescence anisotropy represents a high structural order or low cell membrane fluidity. 

### 2.10. Mitochondrial Reduced (GSH) and Oxidized (GSSG) Glutathione Content

Total glutathione was determined by the glutathione reductase/dithionitrobenzoic (DTNB) method that can measure both GSH and GSSG [51]. Mitochondrial GSH (mGSH) were calculated by subtracting GSSG from the total glutathione content (GSH + GSSG). For this reason samples were run in the presence and absence of 2 mM divinylpyridine.

### 2.11. Programmed Cell Death Biomarkers

#### 2.11.1. Caspase-3 Activity

Caspase-3 activity was measured by a colorimetric assay kit (CASP-3-C), based on the hydrolysis of the synthetic peptide substrate acetyl-Asp-Glu-Val-Asp-p-nitroaniline (Ac-DEVD-pNA) by caspase-3 (Sigma Chem. Co., Buenos Aires, Argentina). The resulting p-nitroaniline (p-NA) released was monitored at 405 nm. Each assay was run in parallel with inhibitor-treated cell lysate (to measure the nonspecific hydrolysis of the substrate) and caspase-3 positive control (using commercial caspase-3, 5 mg/mL provided by the kit manufacturer). A calibration curve using a standard solution of p-nitroaniline (p-NA) was also run for each assay to calculate the activity of the protease expressed as *μ*mol p-NA released/min · mL of sample. 

#### 2.11.2. Bax/Bcl-2 Ratio

Western blot assays of Bax and Bcl-2 were performed to calculate the Bax/Bcl-2 ratio. Equal quantities (30 *μ*g protein per lane) of total protein were separated by SDS-PAGE (15% gels) under reducing conditions and a Miniprotean VI unit (Bio-Rad, CA, USA). The proteins were then electrophoretically transferred to PVDF membranes (IPVH00010 Immobilon, Millipore, USA). The membranes were blocked with 5% skimmed milk and incubated with anti-Bcl-2 and anti-Bax antibodies, respectively (1 : 1000; sc-492 rabbit polyclonal and sc-493 rabbit polyclonal from Santa Cruz CA, USA, resp.), at 4°C overnight. This was followed by incubation with goat anti-rabbit secondary antibody conjugated with horseradish peroxidase (1 : 20.000; Santa Cruz, CA, USA). Photographs were taken using the Molecular Imager Gel Doc XR and ChemiDoc XRS systems (Bio-Rad) and the optical densities of the bands were quantified.

### 2.12. Beta-Amyloid Peptides (1–40) and (1–42)

Beta-amyloid peptides (A*β*) (1–40) and (1–42) were measured using the Human/Rat *β* Amyloid-40 ELISA kit Wako II and the Human Amyloid-42 ELISA kit Wako High-sensitive, respectively. Before the assay the samples were centrifuged (2°C) at 5000 ×g for 15 min and the supernatants diluted 1 : 1 with the buffer provided by the manufacturer. Results for plasma were expressed in pmoles/L and for brain tissues in pmol/mg protein. The A*β*(1–42)/(1–40) ratios were calculated from each individual pair of data. 

### 2.13. Biomarkers of Cardiolipin Peroxidation

#### 2.13.1. Authentic Lipoperoxides

Lipoperoxides (LPOO) in cardiolipin extracts (CL) were determined according to the method of Nourooz-Zadeh et al. [52]. Results were expressed as *μ*moles LPOO/*μ*moles CL (for calculation we used an extinction coefficient of 4.60 (10^−4^) · mol^−1^ · cm^−1^ at 560 nm).

#### 2.13.2. Conjugated Dienes

The spectrophotometric detection of conjugated dienes was performed according to the methodology described by Recknagel and Glende [53]. The spectral register between 300 and 220 nm was obtained and subtracted from a blank spectrum of pure cyclohexane. Conjugated dienes show a typical absorption band in the range of 230–240 nm. The results were expressed as relative units of optical density (UDO)/mg mitochondrial CL.

### 2.14. Statistical Analysis

All values represent the mean of 10 rats assayed in triplicate expressed as mean ± standard deviation (SD). Data were analyzed by ANOVA plus the Tukey test with the aid of SPSS 11.0.1 software (SPSS Inc., Chicago, IL). To analyze the bands obtained by western blot, we used the Image J software for image processing (National Institute of Health, USA). Results were also plotted and analyzed using Sigma Scientific Graphing Software (version 11.0) from Sigma Chem. Co. (St. Louis, MO). The statistical significance (*P* ≤ 0.01) of differences is indicated by superscript letters (values with distinct letters are statistically different between them).

## 3. Results

Growth parameters (final weight gain, rate of body weight, food efficiency ratio, etc.) showed no significant differences between the experimental groups (data not shown). Also, water intake among the experimental groups was essentially identical, which is a reason to assume that the rats did not note any difference in the palatability of the supplemented water compared with the nonsupplemented one. The same happened with solid food.

### 3.1. Copper Content in Mitochondrial Suspensions

Atomic absorption measurements demonstrated that the total Cu content in digested mitochondrial suspensions did not differ significantly among the experimental groups. However, mean values for cortex were higher than those for hippocampus (5.9 ± 0.4 versus 4.0 ± 0.2 g/mg mitochondrial proteins, resp.).

### 3.2. Nonceruloplasmin-Bound Copper (NCBC) and Ceruloplasmin (CP) Concentrations

**Figure 1:**
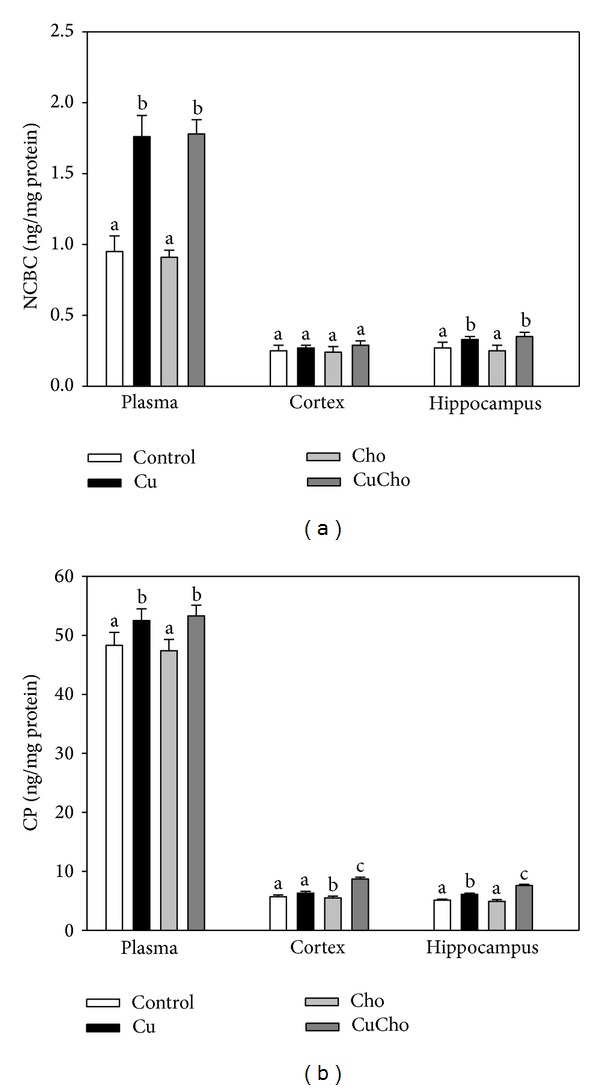
Free Cu (NCBC) (a) and ceruloplasmin (CP) (b) levels in brain cortex and hippocampus homogenates (ng/mg total protein). Treatments are indicated with different colors, control (white bars), Cu (black bars), Cho (gray bars), and CuCho (dark gray bars). Samples were analyzed as indicated in Sections 2.5 and 2.6. Results are expressed as mean of 10 rats assayed in triplicate ± standard deviation (SD). Significant differences among the experimental groups are indicated with superscript letters (values with distinct letters are significantly different between them, *P* < 0.01).

Figure 1(a) shows the concentration of free Cu (expressed as NCBC) in plasma and in both brain zones analyzed. Plasma and hippocampus showed significant increases after Cu and CuCho treatments compared to the control group. In cortex, we observed no significant differences.

We also analyzed CP concentration in plasma, cortex, and hippocampus after the experimental diets (Figure 1(b)). CP increased significantly after Cu and CuCho supplementation in plasma and in both brain zones. Cortex and hippocampus from rats fed on the CuCho diet also showed significant increases with respect to the Cu treatment alone.

### 3.3. Effect of Diets on Mitochondrial Membrane Physicochemical Properties

**Figure 2:**
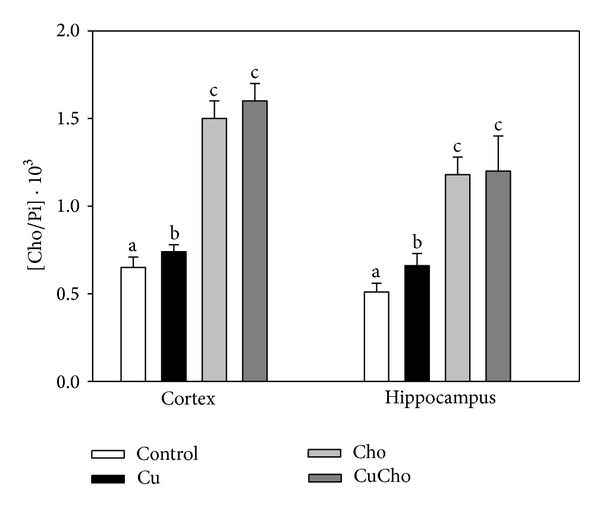
Cholesterol (Cho) and total phospholipids (measured as total inorganic phosphate or Pi) ratio (Cho/Pi) in brain cortex and hippocampus homogenates (ng/mg total protein). Samples were analyzed as indicated in Section 2.7. Treatments are indicated with different colors, control (white bars), Cu (black bars), Cho (gray bars), and CuCho (dark gray bars). Results are expressed as mean of 10 rats assayed in triplicate ± standard deviation (SD). Significant differences among the experimental groups are indicated with superscript letters (values with distinct letters are significantly different between them, *P* < 0.01).

Figure 2 shows the ratio between Cho and total phospholipids (measured as total inorganic phosphate or Pi) in cortex and hippocampus. In both brain zones, Cu treatment produced a significant increase in this ratio. Supplementation with Cho and with Cu + Cho (CuCho) also produced significant increases in the Cho/Pi ratio compared to the control or Cu treatment alone. 

**Figure 3:**
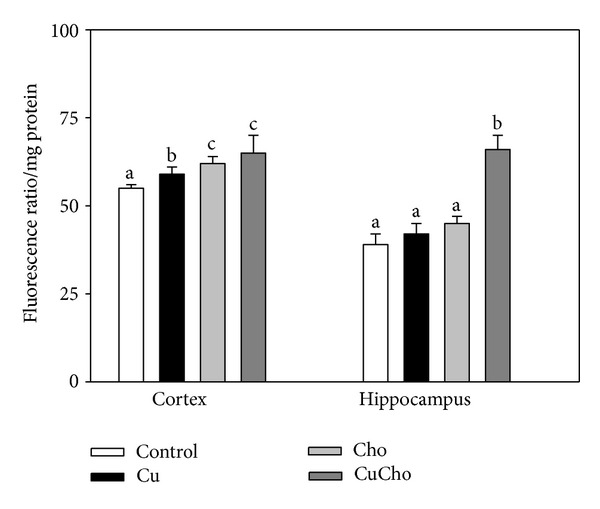
Mitochondrial membrane potential in brain cortex and hippocampus. Treatments are indicated with different colors, control (white bars), Cu (black bars), Cho (gray bars), and CuCho (dark gray bars). Samples were analyzed as indicated in Section 2.8. Results are expressed as mean of 10 rats assayed in triplicate ± standard deviation (SD). Significant differences among the experimental groups are indicated with superscript letters (values with distinct letters are significantly different between them, *P* < 0.01).

The mitochondrial membrane potential was estimated by fluorimetric determination of the degree of aggregation of the JC-1 dye in mitochondrial suspensions from brain cortex and hippocampus (Figure 3). Supplementation with Cu alone produced no significant changes in hippocampus and a discrete although significant increase in the membrane permeability in cortex. In both brain zones cholesterol supplementation also produced a clear loss of integrity which was more pronounced when the lipid was associated with Cu in drinking water (especially in hippocampus). 

### 3.4. Glutathione Content and Biomarkers of Lipid Peroxidation

**Figure 4:**
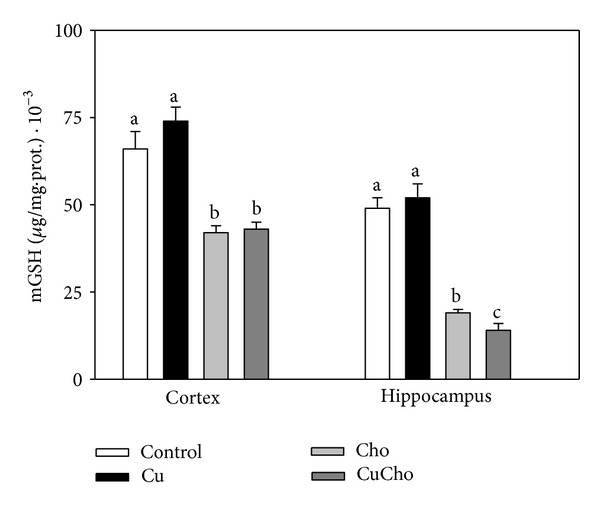
Mitochondrial glutathione (mGSH) content in brain cortex and hippocampus (ng/mg total protein). Treatments are indicated with different colors, control (white bars), Cu (black bars), Cho (gray bars), and CuCho (dark gray bars). Samples were analyzed as indicated in Section 2.10. Results are expressed as mean of 10 rats assayed in triplicate ± standard deviation (SD). Significant differences among the experimental groups are indicated with superscript letters (values with distinct letters are significantly different between them, *P* < 0.01).

Cho and CuCho treatments produced a significant decrease in total mitochondrial glutathione (GSH + GSSG) in both brain zones compared to the control data and to supplementation with Cu alone (Figure 4). Reductions were more evident in hippocampus than in cortex. Addition of inorganic Cu to drinking water did not produce significant changes with respect to the control group.

**Figure 5:**
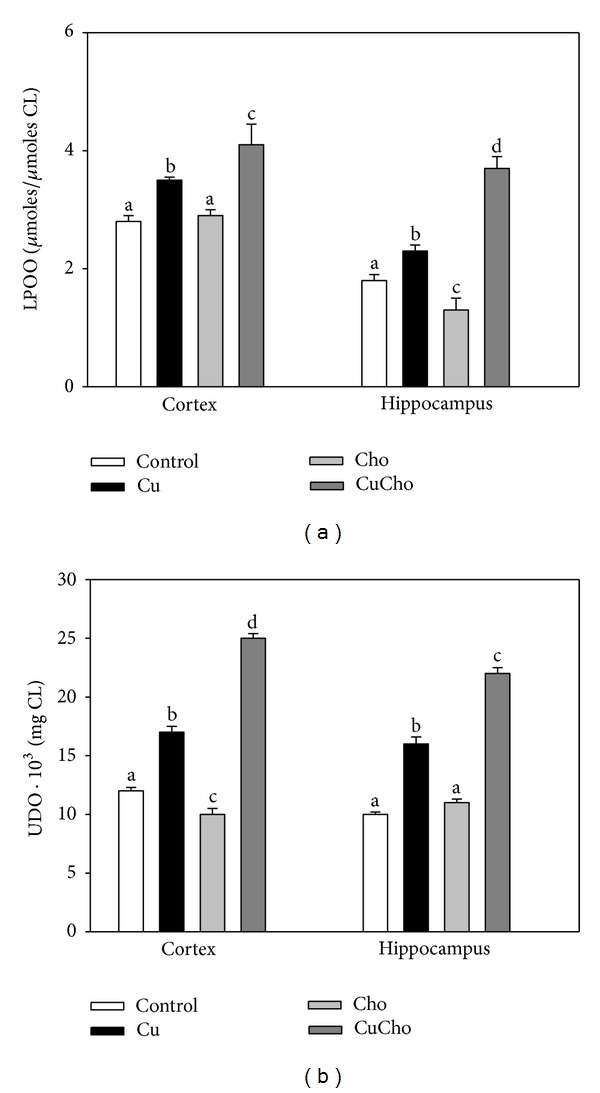
Levels of lipoperoxides (LPOO) (a) and conjugated dienes (b) in mitochondrial fractions from brain cortex and hippocampus. Results are expressed as *μ*moles of LPOO/*μ*Moles of Pi in CL, or UDO/mg protein, respectively. Treatments are indicated with different colors, control (white bars), Cu (black bars), Cho (gray bars), and CuCho (dark gray bars). Samples were analyzed as indicated in Sections 2.13.1 and 2.13.2. Results are the mean of 10 rats assayed in triplicate ± standard deviation (SD). Significant differences among data are indicated with superscript letters (values with distinct letters are significantly different between them, *P* < 0.01).

Figure 5 shows the concentration of two products of CL fatty acids oxidation, lipoperoxides (LPOO), and conjugated dienes (Figures 5(a) and 5(b), resp.). Significant increases in LPOO were observed after Cu addition in hippocampus and in cortex with respect to control data. Cho treatments produced a significant decrease in this biomarker in hippocampus. A very significant increase was observed for the CuCho treatment with respect to supplementation with Cu or Cho alone for both brain zones. After Cu and CuCho treatments, the amount of conjugated dienes was also higher compared to control data in hippocampus and in cortex.

### 3.5. Cardiolipin (CL) and Lyso-Cardiolipin (LCL) Content in Mitochondrial Suspensions

**Figure 6:**
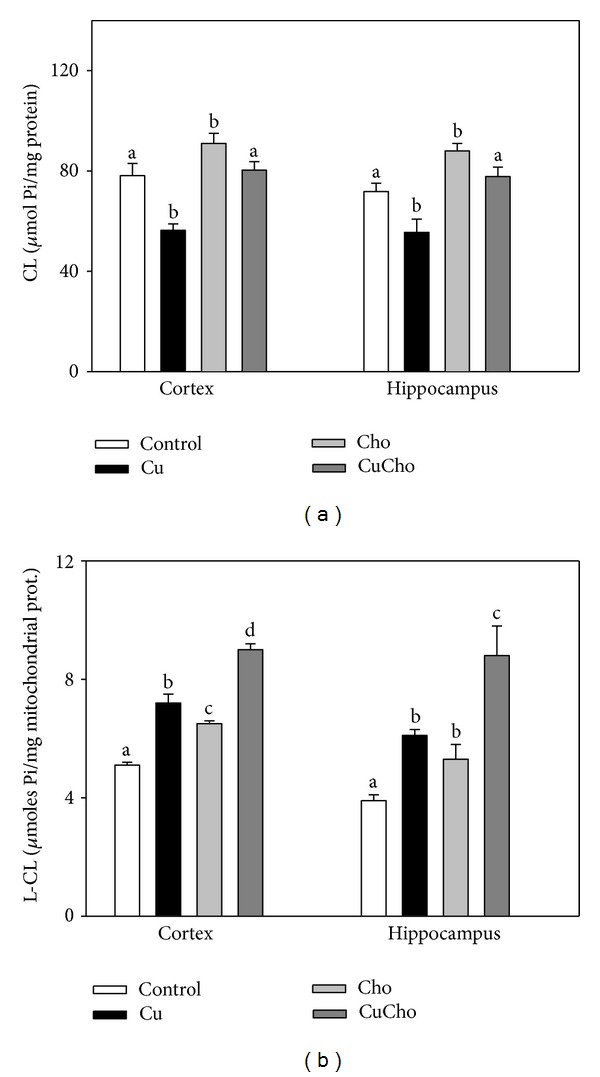
Cardiolipin (CL) (a) and lyso-CL (LCL) (b) levels in mitochondrial fractions from brain cortex and hippocampus (*μ*moles of Pi/mg total protein). Treatments are indicated with different colors, control (white bars), Cu (black bars), Cho (gray bars), and CuCho (dark gray bars). Samples were analyzed as indicated in Section 2.7. Results are the mean of 10 rats assayed in triplicate ± standard deviation (SD). Significant differences among the experimental groups are indicated with superscript letters (values with distinct letters are significantly different between them, *P* < 0.01).

Figure 6(a) shows the total concentration of mitochondrial CL in *μ*mol of inorganic phosphate (Pi)/mg protein. After Cu treatment, CL content decreased in both brain zones analyzed. The Cho-supplemented diet increased CL in both cortex and hippocampus, whereas cosupplementation with Cu and Cho produced no significant changes with respect to the control diet.  Figure 6(b) shows the levels of lyso-CL (LCL) after dietary treatments. All diets induced significant increases in LCL concentrations in hippocampus and cortex compared to control data; however, the higher increases were observed for the groups treated with Cu (alone or in combination with Cho). 

### 3.6. Mitochondrial Membrane Apparent Microviscosities

**Figure 7:**
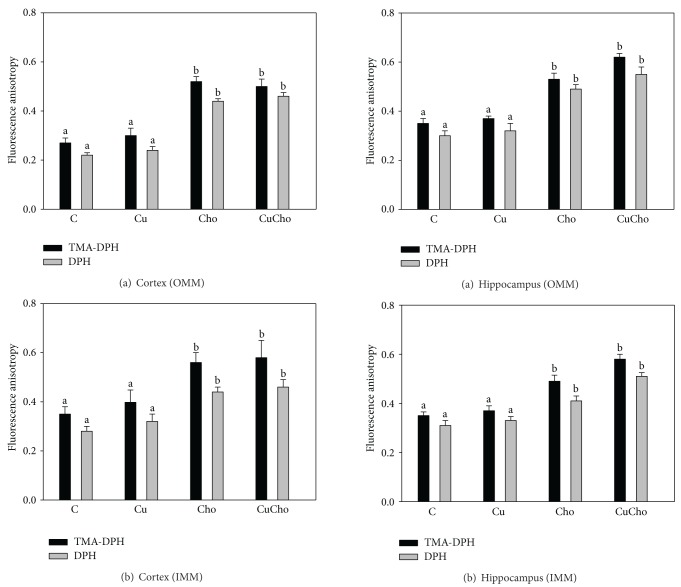
Mitochondrial outer (a) and inner (b) membrane fluidities (fluorescence anisotropy) in suspension isolated from brain cortex. Treatments are indicated with different colors, control (white bars), Cu (black bars), Cho (gray bars), and CuCho (dark gray bars). Samples were analyzed as indicated in Section 2.9. Results are expressed as the mean ± standard deviation (SD) of 10 rats. Significant differences among the experimental groups are indicated with superscript letters (values with distinct letters are significantly different between them, *P* < 0.01).

Isolated outer and inner mitochondrial membranes were examined for their apparent microviscocities using two fluorescent probes (DPH or TMA-DPH) that explore different zones of the lipid bilayer. Figures 7 (cortex) and 8 (hippocampus) show the values of the anisotropies which are inversely correlated with (outer and inner) membrane fluidities. In both brain regions and with both probes tested, we observed decreased fluidities and increased fluorescence anisotropies produced by cholesterol supplementation either alone or in combination with Cu.

### 3.7. Programmed Cell Death Biomarkers

**Figure 9:**
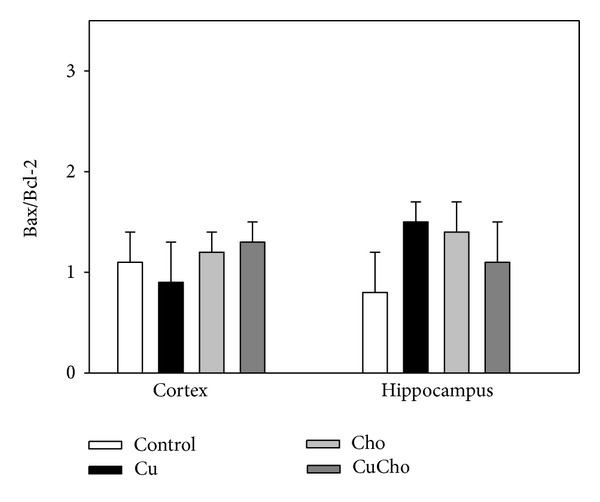
Bax/BcL-2 ratio in brain cortex and hippocampus. Treatments are indicated with different colors, control (white bars), Cu (black bars), Cho (gray bars), and CuCho (dark gray bars). Samples were analyzed as indicated in Section 2.11.1. Results are expressed as the mean ± standard deviation (SD) of 10 rats. There were no significant differences between data.

Despite the damage observed in the integrity of the inner mitochondrial membrane (JC-1 measurements), we did not detect any significant increase in Cyt C in cytosol fractions with the sole exception of a slight—although significant—increase in brain cortex from rats simultaneously treated with Cu and Cho (data not shown). In agreement with this finding, caspase-3 activity was not significantly modified in any experimental group (Table 1) nor was there any significant alteration in the Bax/Bcl2 ratio (Figure 9). 

### 3.8. Biomarker of Neurodegeneration

Table 2 shows the A*β*(1–42)/(1–40) ratio in plasma and in cortex and hippocampus homogenates as a biomarker of neurodegeneration. Cu or Cho treatments produced a significant increase in this biomarker in brain cortex and the CuCho association produced an even more significant increase. Cho supplementation increased the ratio in hippocampus with a value indistinguishable from that observed for the simultaneous treatment with Cu and Cho. These changes were not reflected in peripheral plasma. 

## 4. Discussion

Inappropriate dietary copper and cholesterol intakes are implicated in the development of Alzheimer's disease. This study follows previous experimental evidence reporting the impact of dietary Cu and Cho manipulations as pro-neurodegenerative cause in animal models. 

The impact of elevated inorganic Cu, Cho, or a combination of both in the diet (for 2 months) significantly modifies the mitochondrial function. Results suggest that these dietary supplements are able to increase oxidative damage in brain cortex and hippocampus (with a substantial increment of the lipoperoxides and conjugated dienes in the cardiolipin subfraction), increase the proportion Cho/phospholipids, and consequently decrease the concentration of mGSH, decrease the membrane potential, modify the proportion of cardiolipin and lyso-derivatives, alter inner and outer mitochondrial membrane fluidities, and increase A*β*(1–42)/(1–40) ratio.

It is well known that the metabolism of Cu in humans is under strictly regulated control. The usual concentration of total Cu in human plasma (as determined by us and other groups) is in the range of 0.3 to 2.1 mg/L for intakes of 1.4 to 2.0 mg Cu/day [16]. However, the amount of Cu not bound to ceruloplasmin (NCBC) is very low and has been associated with some of the features of neurodegenerative disorders such as AD [16, 17]. Cholesterol was also associated with the etiology or increased risk of developing AD [54–57]. Thus, our experimental system was designed to investigate the possible concurrence of these two factors in promoting neurodegeneration, with a focus on their role in mitochondrial function. Mitochondrial dysfunction due to ROS overproduction and/or the alteration of the physicochemical properties of their membranes (mainly lipid composition) was reported to be the main factor in the advance of AD, featuring early on in the progression of the disease [25–27].

In discussing the validity and/or limitations of our experimental system, it is necessary to consider the level of the supplementation with Cu and Cho and the characteristics of the metabolism of these two dietary supplements. With respect to Cu overload using oral administration, our experimental conditions were based on previous work [23, 24] and resemble the Cu levels commonly found as a consequence of involuntary exposure through air, food, and water pollution [8, 58–60], ingestion of dietary mineral supplements, and in professionals engaged in agrochemical activities [9–12] or female users of Cu-based intrauterine devices [13, 14]. Studies performed in rats demonstrated that Cu metabolism and homeostasis are essentially identical to those in humans [61]. Similarly, oral Cho supplementation was selected on the basis of previous experiments by other researchers [22–24], and the plasma Cho concentration represents an approximately 30% increase over the medium values obtained for the control group. This increase is frequently observed in humans with dyslipemia [62]. We chose a treatment duration equivalent to approximately 6 years in human terms and therefore assume that the experimental exposure conditions can reasonably be extrapolated to human populations. The known differences between the metabolism of lipoproteins in rats as opposed to humans could be considered a limitation to this assumption, but this can be taken into account as in the case of other experimental models (rabbits or hamsters, e.g.). It is important to recall that rats are quite unique in terms of their sterol metabolism, since in other species studied previously (rabbit, guinea pig, hamster, and squirrel monkey) the liver displays a secondary function, whereas extrahepatic tissues play a quantitatively major role in whole animal sterol biosynthesis and metabolism [63]. 

Cu supplementation in the trace amounts used here did not substantially modify the concentration of the metal inside the mitochondria isolated from cortex or hippocampus. However, after two months of treatment, we observed an increase in CP concentration in both brain zones in those experimental groups that received Cu. We attribute this increase mainly to a response to the proinflammatory condition induced by the Cu-induced oxidative stress as described in our previous work [64]. We were unable to measure the NCBC *inside* the purified mitochondrial fraction mainly due to the lack of CP or to the presence of this protein in amounts beyond detection by our method of measurement. However, in plasma and in whole homogenates we found that NCBC was increased by Cu administration even in the small amounts used here. Concomitantly, the CP concentration was also higher in all samples except cortex, which probably has a strong homeostatic system to control Cu levels. In agreement with previous findings, Das et al. [65] reported that increases of free Cu (NCBC) stimulate the expression of CP through the activation of AP-1. 

Experimental diets also produced significant changes in the lipid composition of mitochondrial fractions. Cu supplementation increased the Cho/phospholipid ratio in the cortex and hippocampus of Wistar rats. This increase was even more pronounced after Cho or CuCho addition. These changes must obviously be associated with the modification of the metabolism (uptake, biosynthesis, and/or degradation) of both subtypes of lipids. The elucidation of these effects remains to be investigated in detail, opening a new avenue of research of potential importance in the search for therapeutic targets in neurodegenerative processes. As a consequence of the aforementioned changes, we observed significant modifications in the physicochemical properties of mitochondrial membranes. The transmembrane potential was altered in Cu- and Cu + Cho-treated rats. Furthermore, the apparent microviscocity of both mitochondrial membranes (outer and inner) was profoundly altered by Cho and CuCho treatments. Generalized and extensive damage in the form of increased permeability (JC-1) and rigidization was mainly demonstrated by the use of two probes with substantially different capacities of lipid bilayer exploration (DPH and TMA-DPH). These findings are in agreement with previously reported data associating the loss of membrane fluidity with an increase in the Cho/Pi ratio [66]. These changes are of relevance from the physiological point of view. It is known that many membrane protein functions including carriers, enzymes, and receptors, among others, can be modulated by the microenvironment of the membrane where they are embedded [66]. Since ROS overproduction has a key role in AD and other neurodegenerative illnesses [67, 68], we will focus on the possible consequences of changes in membrane properties on mitochondrial redox homeostasis and the possible activation of programmed cell death signals.

**Figure 10:**
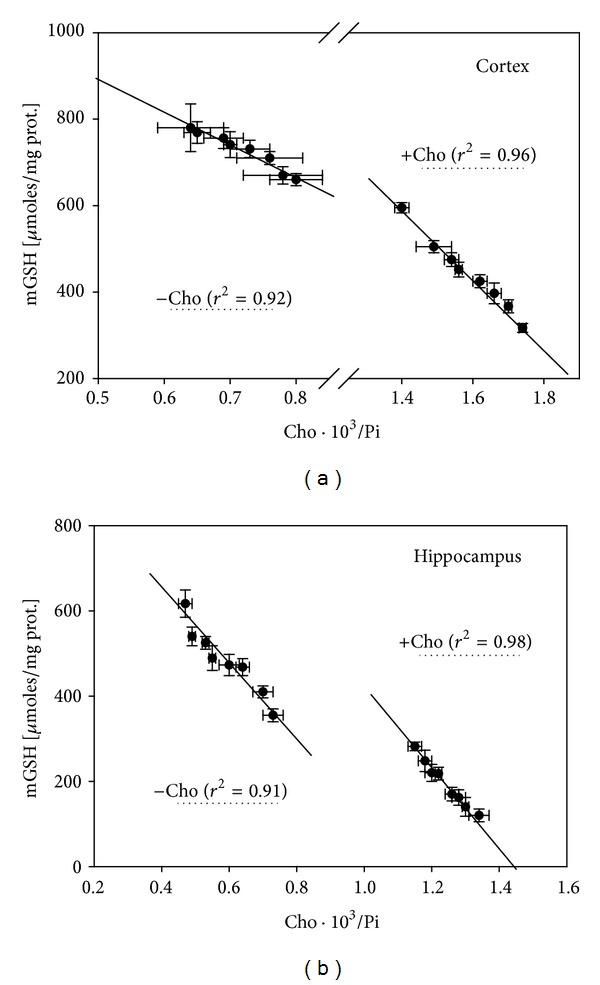
Lineal correlation coefficients for the relationship between mGSH and Cho/Pi ratio in cortex (a) and hippocampus (b) grouped according to two groups of data independently of Cu supplementation, one for rats that did not receive cholesterol (−Cho) and the other for animals fed on diets supplemented with Cho (+Cho). The values of the *r* ^2^ for each curve adjustment appear in parenthesis.

Mitochondrial glutathione (mGSH) is considered the key antioxidant for mitochondrial functioning and survival. It is the main line of defense for the maintenance of the appropriate mitochondrial redox environment by preventing or repairing oxidative modifications that could lead to mitochondrial dysfunction and cell death. The importance of mGSH lies not only in its abundance but also in its extreme versatility to counteract hydrogen peroxide, lipid hydroperoxides, or xenobiotics, mainly as a cofactor of enzymes such as glutathione peroxidase or glutathione-S-transferase. Many cell-inducing stimuli, such as excess NCBC or other transition metals that induce oxidative stress, reduce the levels of mGSH and sensitize the mitochondria to additional insults (e.g., excess cholesterol in the composition of their biomembranes) [66]. Interestingly, the transport of GSH into the mitochondria is critically dependent on the maintenance of physiologically regulated membrane parameters, especially the Cho/Pi ratio and the apparent microviscocity of the inner mitochondrial membrane [66–70]. The dicarboxylate carrier and the 2-oxoglutarate carrier have been shown to function as GSH transporters highly sensitive to the physiochemical state of mitochondrial membranes [69]. The activities of both GSH carriers were inhibited for a decrease in the membrane fluidity. In agreement with these facts, we not only observed a decrease in mGSH in the Cho-supplemented rats but also a strong negative correlation between mGSH and the Cho/Pi ratio in mitochondrial membranes (Figure 10). Cu supplementation also produced an increase in the Cho/Pi ratio, though not sufficiently to significantly modify the membrane fluidity, leaving it unable to modify the mGSH levels. 

Depletion of the main antioxidant defense system in mitochondria is directly linked to increased lipid peroxidation. A pro-oxidative environment clearly promotes the oxidation of CL and also stimulates its regeneration or remodeling activity by increasing the intermediary metabolites, lyso-CL (mono- and dilyso-CL in cortex and hippocampus). In line with this, we found higher LPOO and conjugated diene production in isolated CL subfractions after Cu addition that was even more pronounced after cosupplementation with Cho (CuCho). Peroxidized CL decreases the pool of CL available in mitochondria, thus preventing the formation of the transition pore and the subsequent leakage of Cyt C to the cytosol. Moreover, oxidized CL was able to produce mitochondrial dysfunction, preventing electron transport through the protein complexes [28, 68]. Our finding of lower mitochondrial CL content after Cu treatment in both brains zones is in agreement with previous observations of Yurkova et al. [71]. They reported that Cu ions possess the ability to fragment CL with the subsequent formation of phosphatidic acid and phosphatidyl hydroxyacetone in mitochondria from a mouse model of Wilson's disease. Interestingly, Cho supplementation produced an increase in CL levels probably through an ROS-dependent overstimulation of acyl-transferase-1 activity [72]. The fact that no significant changes with respect to control rats were observed under CuCho treatment could be explained in terms of a compensatory mechanism to counteract the opposite effects produced by Cu and Cho supplementations. 

Cho enrichment of mitochondrial membranes has other important consequences. Alterations in the physical properties of mitochondrial membranes, such as those revealed by the response to DPH and TMA-DPH probes, could modify mitochondrial dynamics, which are critical for the maintenance of mitochondrial integrity and functions including energy production, ROS generation and apoptosis regulation [26–31]. Modifications in mitochondrial dynamics lead to structural changes in cristae formation and the assembly of electron transport complexes, compromising bioenergetics and causing calcium dyshomeostasis, increased oxidative stress (consumption of mGSH), mitochondrial DNA damage, and synaptic dysfunction [28–30].

We have mentioned that the pro-oxidative condition induces CL peroxidation and in consequence diminishes CL's ability join to proteins like Cyt C. Montero et al. [73] and Eckmann et al. [74] emphasized that even though only 15% of the Cyt C is strongly bound to CL, the oxidation of minor amounts of this phospholipid could trigger the mobilization of the Cyt C from the mitochondrial inner membrane to the intermembrane space and then to the cytosol. However, we found no significant changes in Cyt C release to the cytoplasm after any treatments, except in the case of CuCho addition to the cortex. There were also no significant changes in the Bax/BcL-2 ratio and in caspase-3 activity, indicating that the intrinsic apoptotic pathway was still not significantly activated. The pro-oxidative conditions likely induced by Cu, together with the enrichment of cholesterol that reduces mGSH levels, produce CL peroxidation; however, it appears that the level of damage is insufficient to produce a significant stimulation of apoptosome formation and subsequent activation of the caspase-3 pathway [73]. Our lab is now studying the effect of a more prolonged time of exposure in order to investigate the chronic effects of these experimental treatments. 

Lu et al. [24] reported that Cu is associated with A*β* in senile plaques and that this complex can recruit Cho molecules, oxidizing them and producing even more ROS. Despite the clear activation of the proapoptotic cascade, we found an increase in A*β* formation in the brain, a condition which is a recognized biomarker of neurodegeneration. These changes in cortex and hippocampus were not reflected in peripheral plasma, most likely because the latter is less sensitive or the duration of the treatment was not sufficient to affect the proportion between A*β*(1–42)/(1–40) in peripheral plasma, or both. Oxidative stress induces A*β* accumulation in mitochondrial membranes, causing structural and functional damage. Amyloid-*β* interacts with the mitochondrial protein A*β*-binding alcohol dehydrogenase (ABAD) which is upregulated in the temporal lobe of AD patients as well as in A*β*PP transgenic mice [28]. This complex prevents the binding of nicotinamide adenine dinucleotide to ABAD, thereby changing mitochondrial membrane permeability (as we observed in our model) and reducing the activities of respiratory enzymes triggering elevated ROS production [28–30]. Increased A*β* production and A*β*-ROS-dependent formation are linked to other important issues such as the inactivation of the presequence protease (PreP), one of the most important proteins involved in A*β* degradation; alteration of mitochondrial dynamics (previously discussed); increased nitrosative stress; activation of cyclophilin D (an integral part of the permeability transition pore or mPTP); potentiation of synaptic failure; and cytoskeletal aberrations, among others [26–31]. All these facts indicate the importance of monitoring the level of A*β* production not only at the local (SNC) but also at the systemic (peripheral plasma) level.

## 5. Conclusions 

Our work demonstrates that dietary supplementation with trace amounts of inorganic Cu in drinking water + Cho supplementation in solid food causes oxidative stress in brain cortex and hippocampus by depletion of mGSH in an inverse Cho/Pi-dependent fashion; CL peroxidation and stimulation of the remodeling route; major alterations in mitochondrial membrane integrity and fluidity; and a higher A*β*(1–42)/(1–40) ratio as a biomarker of neurodegeneration. These changes may be additive and could lead—under more prolonged exposure—to activation of the proapoptotic cascade. On-going research will shed further light on the real significance of the present results and establish the intimate mechanisms underlying the damage induced by these two very common factors (Cu and Cho overload) with a view to defining potential preventive strategies in human populations at risk.

## Figures and Tables

**Figure 8:**
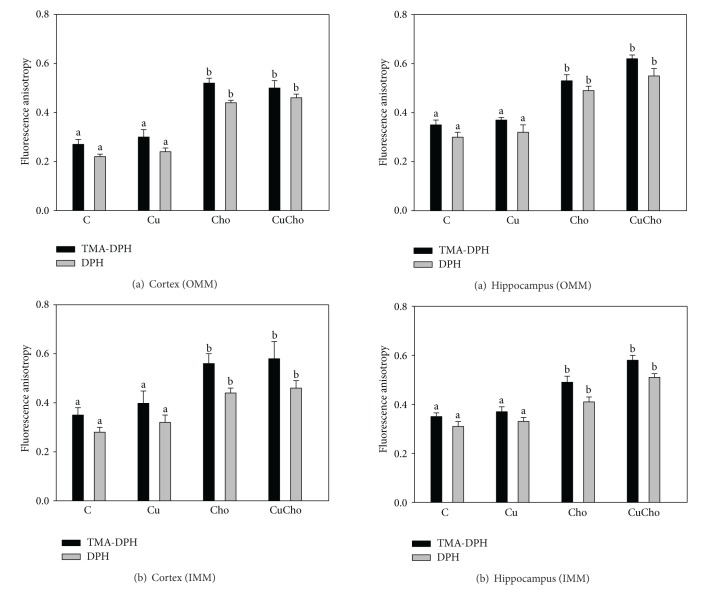
Mitochondrial outer (a) and inner (b) membrane fluidities (fluorescence anisotropy) in suspension isolated from brain hippocampus. Treatments are indicated with different colors, control (white bars), Cu (black bars), Cho (gray bars), and CuCho (dark gray bars). Samples were analyzed as indicated in Section 2.9. Results are expressed as the mean ± standard deviation (SD) of 10 rats. Significant differences among the experimental groups are indicated with superscript letters (values with distinct letters are significantly different between them, *P* < 0.01).

**Table 1:** Caspase-3 activity in brain cortex and hippocampus from the experimental diets.

Diets	Cortex	Hippocampus
C	2.1 ± 0.1	1.4 ± 0.1
Cu	2.3 ± 0.2	1.5 ± 0.1
Cho	2.0 ± 0.1	1.4 ± 0.3
CuCho	2.4 ± 0.1	1.7 ± 0.1

Results were obtained as described in Section 2.11.1. They were expressed as the mean of 10 independent determinations ± SD. There are no statistical differences ascribed to the experimental diet.

**Table 2:** Ratio A*β*(1–42)/(1–40) in plasma and brain cortex and hippocampus after the experimental diets.

Diets	Plasma	Brain	
		Cortex	Hippocampus
C	6.5 ± 1.0^a^	7.3 ± 0.9^a^	6.3 ± 0.7^a^
Cu	6.8 ± 0.8^a^	9.2 ± 0.5^b^	6.6 ± 0.4^a^
Cho	7.1 ± 1.1^a^	9.2 ± 0.6^b^	9.6 ± 0.5^b^
CuCho	8.5 ± 0.8^a^	10.9 ± 0.5^c^	9.3 ± 0.6^b^

Data were obtained as described in Section 2.12. They correspond to the mean of 10 individual determinations assayed in triplicate ± SD. Differences statistically significant among the different diets are indicated with distinct superscript letters.

## Abbreviations

Cu: Copper
CL: Cardiolipin
CP: Ceruloplasmin
Cho: Cholesterol
LPOO: Lipoperoxides
mGSH: Mitochondrial glutathione
ROS: Reactive oxygen species.
